# Small non‐coding RNA and colorectal cancer

**DOI:** 10.1111/jcmm.14209

**Published:** 2019-02-23

**Authors:** Hui Chen, Zhiying Xu, Deliang Liu

**Affiliations:** ^1^ Department of Gastroenterology People’s Hospital of Taizhou Taizhou Jiangsu China; ^2^ Department of Gastroenterology The Second Xiangya Hospital, Central South University Changsha Hunan China

**Keywords:** colorectal cancer, miRNA, piRNA, small non‐coding RNA, tRF

## Abstract

Colorectal cancer (CRC) is the third most common malignance. Although great efforts have been made to understand the pathogenesis of CRC, the underlying mechanisms are still unclear. It is now clear that more than 90% of the total genome is actively transcribed, but lack of protein‐coding potential. The massive amount of RNA can be classified as housekeeping RNAs (such as ribosomal RNAs, transfer RNAs) and regulatory RNAs (such as microRNAs [miRNAs], PIWI‐interacting RNA [piRNAs], tRNA‐derived stress‐induced RNA, tRNA‐derived small RNA [tRFs] and long non‐coding RNAs [lncRNAs]). Small non‐coding RNAs are a group of ncRNAs with the length no more than 200 nt and they have been found to exert important regulatory functions under many pathological conditions. In this review, we summarize the biogenesis and functions of regulatory sncRNAs, such as miRNAs, piRNA and tRFs, and highlight their involvements in cancers, particularly in CRC.

## 1 INTRODUCTION

Colorectal cancer (CRC) is the third most common malignance and is one leading cause of cancer‐related deaths in the world.^1^ Despite great efforts that have been made to understand the pathogenesis of CRC, the underlying mechanisms are still largely unknown. Increasing evidence has demonstrated that CRC is a heterogeneous disease, and its pathogenesis is involved with the activation of oncogenes and inactivation of tumour‐suppressive genes, which are mostly resulting from genetic mutations and epigenetic alterations, the latter including DNA methylation, histone modification and non‐coding RNAs (ncRNAs).^2^, ^3^, ^4^, ^5^, ^6^, ^7^

Large‐scale genome sequencing has indicated that the human genome encodes approximately 20 000 protein‐coding transcripts, which account for only around 2% of the genome, while more than 90% of the total genome is actively transcribed, but lack of protein‐coding potential, referred to as ncRNAs.^8^, ^9^ For several decades, ncRNAs were considered as ‘evolutionary junk’. However, more and more evidence has shown that a part of ncRNAs are functional RNA molecules.^10^ These functional RNA transcripts are composed by housekeeping ncRNAs, including highly abundant ribosomal RNAs (rRNAs) and transfer RNAs (tRNAs), as well as regulatory ncRNAs, such as microRNAs (miRNAs), PIWI‐interacting RNA (piRNAs), tRNA‐derived small RNA (tRFs), small nucleolar RNA (snoRNAs), siRNAs, long non‐coding RNAs (lncRNAs) and circular RNAs (circRNAs). In addition, according to the length of ncRNAs, they are divided into two subclasses: small or short non‐coding RNAs (sncRNAs, 18‐200 nt) and lncRNAs (>200 nt).

Long non‐coding RNAs are a subgroup of non‐coding RNAs, with more than 200 nucleotides in length and no protein coding potential. Long non‐coding RNAs have tissue‐specific expression and exert regulatory functions in many biological and pathological processes. They can function as both tumour suppressors and promoters in CRC development.^11^ For example, H19 is an imprinted oncofoetal ncRNA, but it is hypomethylated and thus up‐regulated in CRC. H19 can promote the development of CRC via generating miRNA or by serving as ceRNA.^12^ Down‐regulation of lncRNA MEG3 can promote colorectal adenocarcinoma cell proliferation and inhibit the apoptosis by up‐regulating TGF‐β1 and its downstream sphingosine kinase 1.^13^ Koduru et al identified differentially expressed lncRNAs in CRC samples. It revealed 18 lncRNAs in tumour vs benign, 89 in metastasis vs benign, and 15 in metastasis vs tumour groups being significantly expressed.^14^

Circular RNAs are another type of RNA with their 3′‐ and 5′‐ends joined together to form a covalently closed loop. They are widely expressed in human cells and have essential roles in the progression of CRC. Circular RNAs can function as a sponge for miRNAs to modulate gene expression by eliminating the inhibitory effect of miRNAs on their target genes. For instance, circRNA hsa_circ_0000523 can regulate the proliferation and apoptosis of CRC cells as miRNA sponge.^15^ Circ‐ZNF609 promotes migration of CRC by inhibiting Gli1 expression via microRNA‐150.^16^

Recent studies have revealed that regulatory sncRNAs (miRNAs, piRNAs, tRFs and snoRNAs) can also function as important regulators in gene expression, and play crucial roles in many physiological and pathological processes. And the abnormal expression of these sncRNAs is involved in many human diseases, including cancers.^17^ In this review, we provide an overview of the representative classes of sncRNAs, including miRNAs, piRNAs and tRFs, and summarize their involvements in CRC, focusing on their roles in the initiation and development of CRC, as well as their biomarker potential (Table 1).

**Table 1:** Dysregulated small non‐coding RNAs in CRC

Up‐regulated	Reference	Down‐regulated	Reference
MicroRNAs			
let‐7b/g	38	let‐7a	56
miR‐7	37	miR‐7	57
miR‐9	39	miR‐9	37
miR‐17	40	miR‐18a^*^	59
miR‐20	41	miR‐26b	60
miR‐21	39, 42	miR‐27b	61
miR‐23a	35	miR‐29b	37
miR‐31	40, 42	miR‐34a	62
miR‐92a	43	miR‐101	63
miR‐96	44	miR‐125	64
miR‐106	84	miR‐138	65
miR‐135	45, 75	miR‐143	66, 67
miR‐141	46	miR‐144	68
miR‐155	47	miR‐145	67, 69
miR‐193a‐3p	35	miR‐194	70
miR‐205	40	miR‐195	71
miR‐214	48	miR‐320a	72
miR‐224	49	miR‐365	73
miR‐338‐5p	35	miR‐491	74
miR‐372	50		
miR‐708	51		
piRNAs			
piR‐651	91	piR‐015551	101
piR‐823	100		
piR‐54878	91		
piR59056	91		
piR‐62701	91		
tRNA‐derived fragments			
tRF‐3^LeuCAG^	116	tRF/miR‐1280	117

piRNA, PIWI‐interacting RNA, tRNA, transfer RNA.

## 2 MICRORNAS

**Figure 1:**
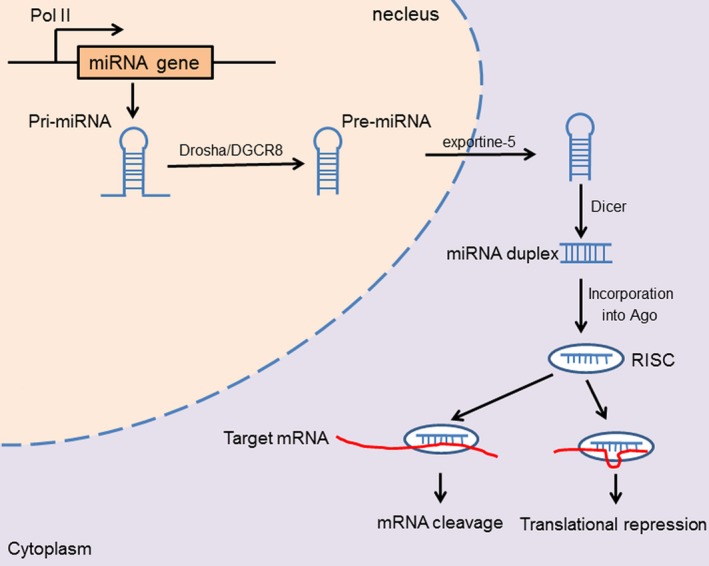
miRNA biogenesis and their functions. A miRNA gene is transcribed by RNA polymerase II (Pol II) to form a primary transcript (pri‐miRNA) with a hairpin loop. The hairpin in the pri‐miRNA is identified by the Drosha‐DGCR8 microprocessor complex, and the pri‐miRNA is converted into the pre‐miRNA. The pre‐miRNA is exported from the nucleus into the cytoplasm by Exportin‐5. Then the pre‐miRNA is processed by the RNase III endonuclease Dicer, yielding a miRNA‐double duplex, which is loaded into Argonaute (Ago) protein to generate the RNA‐induced silencing complex (RISC), then one strand of the duplex is degraded while the other guides the RISC to the target mRNA, thus leading to direct mRNA cleavage or translational repressing

MicroRNAs are a class of sncRNAs containing approximately 18‐25 nucleotides, highly conserved and present in eukaryotic cells. In general, the majority of miRNA‐coding genes are located in intergenic or intragenic regions. The generation of mature miRNAs is a multi‐step process that starts in the nucleus and ends in the cytoplasm. Canonically, most miRNAs are transcribed as large mono‐ or polycistronic primary miRNA precursors (pri‐miRNAs) in the nucleus by RNA polymerase II (Pol. II), while other pri‐miRNAs are transcribed by RNA Pol. III. All the pri‐miRNAs contain a 5′ cap and a poly A tail at the 3′ untranslated region (UTR). Then, pri‐miRNAs are processed to generate miRNA precursors (pre‐miRNAs) by RNase III Drosha in complex with DGCR8, a RNA‐binding protein functioning as a ruler to measure cleavage point. The pre‐miRNAs are subsequently recognized and exported to the cytoplasm by Exportin‐5 (Exp5). In the cytosol, the pre‐miRNAs are cleaved by the RNase III endonuclease Dicer to yield a miRNA duplex, which contains a leading strand or miR and a passenger strand or miR*. The functional strand is loaded into the RNA induced silencing complex (RISC), while another one is destroyed following the attachment to argon‐binding proteins (AGO). miRNAs can negatively regulate gene expression in a post‐translational manner. Generally, after being loaded into RISC, the functional miRNAs recognize and basepair with specific seed sequences within the 3′ UTR of target mRNAs, resulting in direct mRNA degradation or translational silencing. When miRNAs are almost completely complementary with their mRNA targets, the targeted mRNA can be directly cleaved and degraded. However, the overwhelming majority of miRNAs and their target mRNAs are only partially complementary. In this case, the non‐perfect pairing manner, which usually involves a seed pairing of just six to seven nucleotides in length, often leads to the transcription inhibition (Figure 1).^18^ Additionally, recent studies have shown that miRNAs may also positively regulate gene expression. miR‐10a binds to the 5′ UTR of ribosomal protein mRNAs and promotes their translation.^19^ miR‐21 directly targets mitochondrial cytochrome b (mt‐Cytb) and enhances mt‐Cytb translation.^20^

As novel regulators of gene expression, miRNAs have a profound impact on the initiation and development of many cancers.^17^, ^21^, ^22^ A series of studies have revealed that miRNAs can act as oncogenes called ‘Oncomir’ or tumour suppressor, thus exerting an important role in many biological behaviours of cancer cells, involved with cell proliferation, apoptosis, metastasis and drug resistance. Furthermore, aberrant miRNAs can also be used as a potential diagnostic and prognostic biomarker for cancers.^17^, ^23^, ^24^, ^25^, ^26^, ^27^, ^28^ Recently, the involvement of miRNAs in the pathogenesis of CRC has been relatively clarified.^7^, ^29^, ^30^, ^31^, ^32^

By microarray‐based miRNA profiling platforms and next‐generation sequencing (NGS) approaches, a number of differentially expressed miRNAs have been identified in CRC. In 2003, Michael et al firstly reported some altered expressed miRNAs, among which, miR‐143 and miR‐145 were found to be reduced in CRC tissue compared to healthy tissue.^33^ In another study, Schetter et  al screened 37 differentially expressed miRNAs by miRNAs microarray in 84 CRC tissues.^34^ In addition, a panel of dysregulated miRNAs has been detected by Real‐time PCR in CRC. Yong et  al identified some differentially expressed miRNAs in both CRC tissues and blood derived from CRC patients, of which, miR‐23a, miR‐193a‐3p and miR‐338‐5p were significantly up‐regulated in CRC tissues, and the levels of these miRNAs are positively related between CRC tissue and blood.^35^ Meanwhile, an exosomal miRNA profile was used to highlight the most abundant miRNAs in the blood of 88 patients with CRC.^36^ As well as circulating miRNAs, some stool‐based miRNAs were also observed to be dysregulated and they also can be used as a biomarker for CRC. For example, miR‐7, miR‐17, miR‐20a and miR‐21 are increased in the stool of CRC patients, while miR‐9, miR‐29b, miR‐138 and miR‐143 are decreased.^37^

Dysregulated miRNAs have significant effects on the biology of cancer cells, and they can act as oncogenes or tumour suppressor genes, therefore contributing to the development of CRC. Typically, oncogenic miRNAs are up‐regulated in CRC and often target endogenous tumour suppressors. Some of these miRNAs are let‐7b and 7 g,^38^ miR‐9,^39^ miR‐17,^40^ miR‐20,^41^ miR‐21,^39^, ^42^ miR‐31,^40^, ^42^ miR‐92a,^43^ miR‐96,^44^ miR‐135,^45^ miR‐141,^46^ miR‐155,^47^ miR‐205,^40^ miR‐214,^48^ miR‐224,^49^ miR‐372^50^ and miR‐708.^51^ For example, miR‐21, which has been proven as an oncogenic miRNA in several cancers, can promote the proliferation and metastasis of CRC cells by targeting some key tumour suppressive genes, including PDCD4, TIAM1, SPRTY, PTEN, TGFBR2 and CDC25A.^52^, ^53^, ^54^, ^55^, ^56^ miR‐92a has been shown to promote the epithelial‐mesenchymal transition (EMT) process through the suppression of E‐cadherin in CRC.^43^ miR‐96 is up‐regulated in CRC and has been shown to target p53 inducible nuclear protein 1 (TP53INP1), exerting suppressive effects on p53 activity.^44^ miR‐224 has also recently been found to enhance the metastasis of CRC through its modulation of SMAD4.^49^ On the other hand, tumour‐suppressive miRNAs are commonly down‐regulated in CRC tissues, and play a crucial role in down‐regulating oncogenes in CRC. These miRNAs are let‐7a,^57^ miR‐7,^58^ miR‐18a*,^59^ miR‐26b,^60^ miR‐27b,^61^ miR‐34a,^62^ miR‐101,^63^ miR‐125,^64^ miR‐138,^65^ miR‐143,^66^, ^67^ miR‐144,^68^ miR‐145,^67^, ^69^ miR‐194,^70^ miR‐195,^71^, miR‐320a,^72^ miR‐365^73^ and miR‐491.^74^ miRNAs let 7a, miR‐143, miR‐18a* and miR‐145 suppress the development of CRC by down‐regulating RAS.^57^, ^59^, ^66^, ^69^ In addition, miR‐143 and miR‐145 also exert anti‐tumour roles through their down‐regulation of insulin‐like growth factor 1 receptor (IGF1R).^67^ miR‐195 and miR‐491 have been found to promote apoptosis in CRC cells through the targeting of B‐Cell CLL/Lymphoma 2 (BCL2) and BCL2‐Like 1 (BCLXL), respectively.^71^, ^74^

The onset and development of CRC are involved with the activation of many oncogenic signalling pathways, such as Wnt, Ras, TGF‐β and inflammatory signalling pathways. And these signalling pathways can be regulated by miRNAs.^7^ Wnt signalling is one of the most frequently changed pathways in CRC. Previous studies have revealed that miRNAs play an important role in regulating the Wnt signalling pathway through targeting the key molecules of the Wnt pathway. miR‐135a/b is highly expressed in CRC and is able to directly target APC, resulting in the reduction in APC expression and in the up‐regulation of Wnt signalling. miR‐135a/b is also predicted to target secreted frizzled‐related protein 4 (SFRP4), which is a Wnt/β‐catenin inhibitor. Meanwhile, recent studies have showed that miR‐135b can be transcriptionally activated by β‐catenin/TCF4, which leads to the up‐regulation of miR‐135b in CRC.^45^, ^75^ Unlike miR‐135, miR‐21 can increase β‐catenin nuclear translocation and promote tumorigenesis in CRC.^76^ miR‐155 is also a Wnt/β‐catenin stimulator by targeting HMGB1 and indirectly increasing Wnt/β‐catenin expression.^77^, ^78^ The RAS gene family, such as NRAS, HRAS and KRAS, and KRAS has been frequently mutated in CRCs. The dysregulation of KRAS family has been shown to be well‐associated with poorer outcomes, in terms of shorter survival times, and being more aggressive and drug‐resistant. The RAS genes have several miRNA let‐7 binding sites at their 3′ UTR. The reduction in let‐7 in cancer tissues is correlated with higher KRAS mRNA expression; and let‐7 miRNA suppressed colon cancer growth and proliferation.^79^ miR‐143 has been also shown down‐regulated in CRC tissues, and reduced expression of miR‐143 led to cell proliferation in vitro, which is linked to the increased expression of KRAS.^66^ In contrast, miR‐31 has been shown to negatively regulate KRAS inhibitor RASA1; thus, miR‐31 could be a potent enhancer of KRAS in CRC. miRNAs also play important roles in regulating TGF‐β signalling.^80^, ^81^ The miR‐17 family has a crosstalk with the TGF‐β signalling pathway. The miR‐17 family has eight miRNAs, including miR‐17, miR‐18a/b, miR‐20a/b, miR‐93 and miR‐106a/b. miR‐17 targets and inhibits PTEN and RHOE (RND3).^42^, ^82^ miR‐20a promotes cancer progression by facilitating CRC cell line migration and invasion and up‐regulating the expression of EMT markers.^83^ miR‐106a/b seems also to enhance EMT and metastasis by targeting TGF‐β receptor TGFBR2.^84^

## 3 PIWI‐INTERACTING RNAS

P‐element induced wimpy testis (PIWI)‐interacting RNAs are a class of small ncRNAs of 26‐31 nucleotides, which are defined because of their interaction with PIWI subfamily of Argonaute proteins.^85^, ^86^, ^87^ PIWI‐interacting RNA shows a very diverse set of nucleotide sequences compared with other known cellular RNA family members. According to their origin, piRNAs are classified into three groups: transposon‐derived piRNAs, mRNA‐derived piRNAs and lncRNAs‐derived piRNAs.^88^, ^89^, ^90^ PIWI‐interacting RNA are mostly transcribed as large single‐stranded transcripts, which are then exported to the cytoplasm and processed into mature piRNAs independently from Dicer. It is currently known that two mechanisms are involved in the generation of mature piRNAs: the primary synthesis mechanism and the ‘ping‐pong’ amplification mechanism. First, the primary transcript is cleaved by the riboendonuclease Zucchini, and the 3′ fragment of the product is incorporated into PIWI proteins and trimmed by a 3′ to 5′ exonuclease. Then, the 2′ hydroxy group at the 3′ end is methylated by the enzyme Hen1. The piRNA/PIWI complex migrates back to the nucleus, which reaches their target transcripts and mobilizes the silencing machinery to block the transcription of transposable elements, maintaining genome integrity. In addition, the ‘ping‐pong’ amplification mechanism is involved in the process of piRNAs' accumulation. As well as migrating into the nucleus, piRNAs can also bind with AGO3 or AUB proteins to form piRNA/Ago or piRNA/Aub complexes in the cytoplasm, which contain complementary sequences and provide substrate to each other, therefore producing new antisense piRNAs. The ping‐pong framework has been identified in zebrafish, D. melanogaster and other various species. However, previous data have revealed that piRNAs biogenesis during adult spermatogenesis in mice is independent of the ping‐pong mechanism.^88^, ^89^, ^90^ Therefore, the biogenesis of piRNAs is still not clear in mammals, which needs further investigation. Actually, the functions of piRNAs are still not perfectly clear. Up to date, piRNAs have been shown to be involved in transposon silencing, epigenetic regulation, gene and protein regulation, genome rearrangement, spermatogenesis and germ stem‐cell maintenance.^88^, ^89^, ^90^ As reviewed above, piRNAs can bind with PIWI proteins and direct them to their transposon targets, therefore contributing to genetic diversity and genetic instability. Additionally, PIWI can serve as an epigenetic activator and so, as a partner of PIWI proteins, piRNAs are likely to be implicated in transcriptional silencing or activation. PIWI‐interacting RNA is also found to regulate gene expression and protein stability. For example, piRNAs can regulate the ubiqutination level of mouse PIWI protein MIWI by enhancing MIWI interaction with an APC/C substrate‐binding subunit. Recently, some piRNAs have been found to be dysregulated in tumour tissues, and altered piRNAs play an important role in cancer cell proliferation, apoptosis and metastasis, and they may become potential prognostic and diagnostic biomarkers during cancer development.^17^, ^91^ Previous studies have shown that piR‐34377, piR‐34736, piR‐36249, piR‐35407 and piR‐36318 are significantly down‐regulated, while piR‐651, piR‐932, piR‐4987, piR‐20365, piR‐20485, piR‐20582, piR‐36743, piR‐36026, piR‐31106 and piR‐021285 are up‐regulated in breast cancer.^92^, ^93^ piR‐651, piR‐32105, piR‐58099 and piR‐59056 are increased in gastric cancer, whereas piR‐823 is decreased.^94^, ^95^ piR‐55490 is down‐regulated in lung cancer, while piR‐651 is up‐regulated^96^, ^97^; piR‐55490 inhibits lung cancer cell proliferation and the mechanism is associated with its binding to 3′ UTR of mTOR messenger RNA (mRNA).^97^ piR‐32051, piR‐39894 and piR‐43607 were found up‐regulated in kidney cancer tissues.^98^ PIWI‐interacting RNA DQ594040 is one down‐regulated piRNA in bladder cancer, and its overexpression can inhibit bladder cancer cell proliferation, colony formation and promote cell apoptosis through up‐regulating TNFSF4 protein.^99^ As for CRC, piR‐651 was found to be up‐regulated in tumour tissues, which is associated with metastasis state. piR59056, piR‐54878 and piR‐62701 are also highly expressed in CRC, and are associated with recurrence‐free survival.^91^ piR‐823 contributes to colorectal tumorgenesis by enhancing the transcriptional activity of HSF1.^100^ Chu et  al observed that an lncRNA, LNC00964‐3, which includes the piR‐015551 sequence, is significantly lower in CRC tissues. Meanwhile, the expression of piR‐015551 is positively correlated with LNC00964‐3, indicating that piR‐015551 might be generated from LNC00964‐3, and may be implicated in the development of CRC.^101^

## 4 TRNA‐DERIVED FRAGMENTS

**Figure 2:**
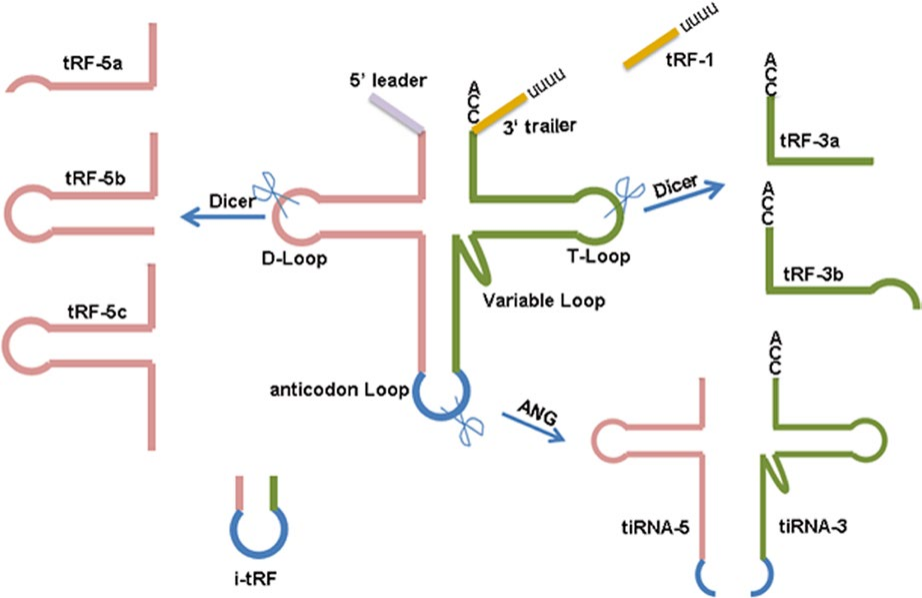
The types of tsRNAs are classified by size and sequence location in the tRNA structure. Based on the length and cleavage sites of tRNAs, small non‐coding RNA derived from tRNAs can be classified into two major groups: tRNA‐derived stress‐induced RNA (tiRNAs), with the length of 28‐36 nt, and tRNA‐derived fragment (tRFs), about 14‐30 nt length

Transfer RNAs are a group of ncRNAs that function as a fundamental component of the translation machinery, which can help deliver amino acids to the ribosome. Traditionally, tRNAs have been considered as key actors in protein synthesis.^102^, ^103^ However, recent studies have revealed that a mass of non‐coding small RNAs are derived from tRNAs, namely which serve as novel regulators in some pathophysiologic processes.^104^ On basis of the length and cleavage sites of tRNAs, small non‐coding RNA derived from tRNAs can be classified into two major groups: tRNA‐derived stress‐induced RNA (tiRNAs), also named tRNA‐halves, produced by specific cleavage of the anticodon loops of mature tRNAs, with the length of 28‐36 nt^105^, ^106^; tRNA‐derived fragment (tRFs), about 14‐30 nt length, derived from the mature or primary tRNAs (Figure 2).^107^ Herein, we will focus on the roles of tRFs in the development of CRC.

tRNA‐derived fragments have three subtypes: tRF‐5s, tRF‐3s and inter tRFs.^107^, ^108^ The tRF‐5s are usually formed by a cleavage in the D‐loop or arm region between D‐loop and anticodon loop of a mature tRNA. The tRF‐3s are generated from a cleavage in the T‐loop, with an end trinucleotides ‘CCA’. The inter tRFs, also termed as tRF‐1s, originate from internal region of mature tRNA, which includes anticodon loop and part of D‐loop and T‐loop.^109^ Based on the cleavage site and sequence length, these fragments are further sub‐classified: tRFs‐5a (14‐16 nt), tRFs‐5b (22‐24 nt), tRFs‐5c (28‐30 nt), tRFs‐3a (18 nt) and tRFs‐3b (22 nt) and most tRF‐1s identified in human cells are 20 and 36 nt in length.^109^ The production of tRF‐5s and tRF‐3s has reported to be dependent on Dicer.^109^ However, the specific process of generating tRF‐5s and tRF‐3s is still not clear. Meanwhile, the biogenesis mechanism of tRF‐1s is also unknown. Recently, tRFs have been found to play an important role in many biological and pathological processes, and they are associated with many diseases, such as virus infection, metabolic disorder, neurodegenerative diseases and cancers.^17^, ^109^, ^110^ To date, the regulatory mechanisms of tRFs mainly involve several strategies as follows. tRNA‐derived fragments have also been reported to be involved in global translational inhibition in human cells. A 19‐nt long tRF‐5 derived from tRNA^Gln ^down‐regulates the expression of a reporter gene, but which does not have a reverse complementary target sequence, suggesting that the translational repression mediated by this tRF‐5 is non‐specific. The translational inhibition of tRFs required a conserved ‘GG’ dinucleotide for their activity, and it may exert the function through interacting with the ribosome.^111^ In addition to global translational inhibition, some tRFs mediate gene silencing similarly to miRNAs.^112^ For instance, upon infection of respiratory syncytial virus (RSV), tRF‐5^GluCTC^ is drastically induced and exhibits a gene‐silencing role by targeting mRNA.^113^

Recently, a series of tRFs have been shown to be altered in cancer tissues, and they are implicated in the development of cancers. tRF‐1001, one of tRF‐1s, is highly expressed in prostate cancer cells and other several cancer cell lines. Knockdown of tRF‐1001 can repress the proliferation of prostate cancer cells, which is associated with DNA synthesis suppression and G2 stage arrest.^107^ CU1276, a tRF‐3 derived from tRNA(Gly), was firstly identified in human mature B cells. Overexpression of CU1276 in lymphoma cells can suppress cell proliferation. Meanwhile, lymphomas cells with CU1276 knockdown are sensitive to DNA damage, resulting in an accumulation of mutations and genomic aberrations during tumour development.^17^ By small RNA‐sequencing of breast cancer cells, it was found that inter tRFs derived from mature tRNA(Glu), tRNA(Asp), tRNA(Gly) and tRNA(Tyr) are induced under hypoxic stress and these inter tRFs are also found to repress cell growth under serum‐starvation, cancer cell invasion and metastasis in breast cancer cells.^114^ The tRF‐3^ThrTGT^ is significantly down‐regulated in primary breast cancer and metastatic tumours; overexpression of tRF‐3^ThrTGT^ in breast cancer cells remarkably inhibits cell invasion and migration.^115^ tRF‐3^LeuCAG^, identified in HeLa and HCT‐16 cells, can promote cell viability.^116^ In CRC, tRF/miR‐1280 was found to be decreased in tumour tissues. The overexpression of tRF/miR‐1280 can reduce cell proliferation and colony formation, whereas knockdown of it would reverse these effects. tRF/miR‐1280 reduces tumour formation and metastasis by directly targeting the Notch ligand JAG2. Interestingly, Notch signalling pathways are essential for cancer stem‐like cells (CSC) in CRC progression. Therefore, tRF/miR‐1280 suppresses CRC growth and metastasis by repressing Notch signalling pathways that support CSC phenotypes.^117^

## 5 CONCLUSION

Although great achievements have been made in understanding the pathogenesis of CRC, the underlying mechanisms are still needed for further investigations. For decades, many studies have provided a lot of evidence that sncRNAs are important regulatory molecules and play a crucial role in cancer development and progression. So, it is believed that sncRNAs are involved in the pathogenesis of CRC. Notably, many miRNAs have been demonstrated to be dysregulated in CRC, and they may exert important functions in the development of CRC. Furthermore, they have been found to act as potential diagnostic biomarkers for CRC. In addition, some piRNAs and tRFs have also been proven to be implicated in the development of CRC. However, studies on them are still at a very early stage, and many questions still need to be addressed. In conclusion, considering sncRNAs as important regulators in CRC, we need to shed light on many aspects of their biogenesis and functions in CRC.

## CONFLICT OF INTEREST

The authors declare no conflict of interest.
